# Self-Compassion, Emotion Regulation and Stress among Australian Psychologists: Testing an Emotion Regulation Model of Self-Compassion Using Structural Equation Modeling

**DOI:** 10.1371/journal.pone.0133481

**Published:** 2015-07-24

**Authors:** Amy L. Finlay-Jones, Clare S. Rees, Robert T. Kane

**Affiliations:** School of Psychology and Speech Pathology, Curtin University, Perth, Australia; Tilburg University, NETHERLANDS

## Abstract

Psychologists tend to report high levels of occupational stress, with serious implications for themselves, their clients, and the discipline as a whole. Recent research suggests that self-compassion is a promising construct for psychologists in terms of its ability to promote psychological wellbeing and resilience to stress; however, the potential benefits of self-compassion are yet to be thoroughly explored amongst this occupational group. Additionally, while a growing body of research supports *self-compassion* as a key predictor of psychopathology, understanding of the processes by which self-compassion exerts effects on mental health outcomes is limited. Structural equation modelling (SEM) was used to test an emotion regulation model of self-compassion and stress among psychologists, including postgraduate trainees undertaking clinical work (*n* = 198). Self-compassion significantly negatively predicted emotion regulation difficulties and stress symptoms. Support was also found for our preliminary explanatory model of self-compassion, which demonstrates the mediating role of emotion regulation difficulties in the self-compassion-stress relationship. The final self-compassion model accounted for 26.2% of variance in stress symptoms. Implications of the findings and limitations of the study are discussed.

## Introduction

Effective stress management has been identified as an ethical imperative among psychologists, with evidence that as many as 40–73% of trainee and professional psychologists report “caseness” levels of distress [1–3]. Stress among psychologists has been linked with a range of negative outcomes, including self-esteem difficulties [4], depression and anxiety [5, 6], secondary traumatic stress [7, 8], compassion fatigue [9–11], and vicarious traumatization (e.g., [7, 12, 13]). Moreover, stress has been found to undermine attention [14] and decision-making skills [15] and may negatively impact therapists’ capacities to empathize with and support their clients [16–18]. In light of these findings, there has been a call for the development of initiatives that promote stress resilience and wellbeing among psychologists in a positive, integrated, and sustainable way [19, 20]. Mindfulness-based interventions have received growing attention in this regard [20–22], and programs such as mindfulness-based stress reduction (MBSR) and acceptance and commitment therapy (ACT) have been found to decrease stress, negative affect, self-doubt and anxiety amongst trainee psychologists and allied health professionals [2, 23, 24]. More recently, self-compassion has been identified as a key target variable in this line of work (e.g., [2, 21, 23, 24, 25]), with some researchers proposing that self-compassion is a central mechanism by which mindfulness- and acceptance-based interventions impact psychological health [26–28].

Preliminary theory and evidence suggests that relating to oneself with compassion is a promising means of promoting self-care, professional wellbeing, and resilience to stress among health professionals (e.g., [19, 29, 30, 31]), however, research in this area is sparse. In addition, understanding of the working mechanisms underlying the link between self-compassion and psychological health is limited. We sought to address this gap in the literature by investigating the relationship between self-compassion and stress symptoms among a sample of Australian psychologists, and testing a preliminary explanatory model of self-compassion, which posits that emotion regulation difficulties mediate the link between self-compassion and stress. Below we present an overview of the research that describes the nature of psychologists’ experience of stress, the link between self-compassion and stress among psychologists and the wider population, and the rationale for exploring emotion regulation difficulties as a mediating mechanism in the relationship between self-compassion and stress.

### Self-Compassion and Psychological Health

Self-compassion is conceptualized as an adaptive form of self-relation that involves three primary capacities: cultivating mindful awareness of one’s own suffering; treating oneself with understanding and kindness during times of difficulty; and relating one’s stressful experiences to the wider perspective of human experience [32]. A growing body of literature documents the association between self-compassion and positive psychological outcomes such as happiness, optimism, contentedness, wisdom, emotional intelligence, and adaptive coping [33–36]. Individuals who are higher in self-compassion report more empathic concern, perspective taking, forgiveness and altruism [37], and appear to have improved relationship functioning [38, 39]. Self-compassion has also been found to negatively predict markers of psychological distress such as neurotic perfectionism, rumination, and thought suppression [40, 41]. Moreover, self-compassion is consistently associated with lower levels of depression and anxiety, with a recent meta-analysis reporting a large effect size (*r* = −0.54) for the relationship between self-compassion and depression, anxiety, and stress [42].

Of particular relevance to the current study are findings that self-compassion appears to attenuate specific acute, chronic and traumatic stress outcomes. For example, self-compassion has been found to predict burnout among clergy members [43] and infertility-related stress in women [44], as well as mediating reductions in symptoms of post-traumatic stress disorder (PTSD) following treatment [45]. Self-compassion may be particularly useful in circumstances involving social evaluative threat—that is, situations in which an aspect of one’s self is at risk of being judged negatively. In a series of studies, Leary, Tate (46) found that both trait and induced self- compassion were associated with reduced emotional reactivity, less negative affectivity, more acceptance, and a greater capacity to place events into perspective among individuals when prompted to recall and imagine real and perceived failures. In addition, a study by Arch, Brown (47), found that participants undergoing brief self-compassion training displayed biopsychosocial and affective responses to social threat that were consistent with lower stress, relative to attention-training and no-training controls. Consistent with this, trait self-compassion has been found to moderate inflammatory responses to laboratory-based social stressors [48, 49].

### Mechanisms of Action

Given the burgeoning interest in self-compassion as a key construct supporting mental health, the dearth of studies investigating the mechanisms by which it impacts psychological health is surprising. In one of the few studies that explored factors mediating the link between self-compassion and psychopathology, Raes [50] found that rumination and worry partially explained the relationship between self-compassion and anxiety, while the relationship between self-compassion and depression was partially accounted for by rumination only. Another study examining the relationship between self-compassion and depression found that the relationship between these variables was mediated by rumination and avoidance [51]. While rumination, worry, brooding, and avoidance all represent specific maladaptive coping strategies [52, 53], we believe that self-compassion is inversely related to problematic emotion regulation more generally. The current study examines self-compassion in the context of Gratz and Roemer’s multidimensional model of emotion regulation difficulties [54] to explore the hypothesis that self-compassion lowers vulnerability to stress by reducing problematic emotion regulation in the face of difficult events and negative affective experiences.

Emotion regulation refers to the ways in which individuals attend to and appraise their emotions as well as the ways they modulate the intensity and duration of emotional states [55, 56]. In the mental health context, maladaptive emotion regulation has been regarded as a transdiagnostic process that underlies a wide range of psychological symptoms and clinically relevant behaviours [52, 56]. While a number of different emotion regulation taxonomies exist [57], one conceptualization of problematic emotion regulation that has garnered significant support is Gratz and Roemer’s [54] multidimensional model. According to this model, individuals who have difficulties with emotion regulation may have problems recognizing, understanding or accepting certain emotional states, and may struggle to access adaptive coping strategies, and to control impulsive behavior and maintain goal-directed behavior in the face of difficult emotional experiences [54]. They may also be more likely to engage in problematic emotion regulation strategies such as rumination [58] and avoidance [54]. These strategies tend to maintain or increase negative affective experiences over time [59, 60] and are consistently implicated in a wide range of psychopathology [52].

There is evidence to suggest that self-compassion positively impacts psychological health by promoting adaptive emotion regulation in times of stress. Researchers have theorized that self-compassion defuses negative emotional patterns by promoting non-judgmental awareness of one’s emotions and orienting oneself to respond to stressful events in a way that is self-supportive [34, 35]. Self-compassionate individuals are more likely to view stressful events within the wider spectrum of human experience, a perspective thought to enhance a sense of relatedness and reduce the feelings of isolation and disconnection that are commonly experienced during times of failure or difficulty [35]. This supports a mindful approach to one’s difficult experiences, in which negative emotions are held in a balanced and non-judgmental way, rather than amplified through processes of over-identification or self-criticism [32]. Mindful awareness is an established predictor of adaptive emotion regulation and coping [52, 61], and it has been suggested that self-compassion may promote healthy emotional responding in similar ways [32]. Importantly, self-compassion goes beyond responding to difficult emotions with equanimity, by actively encouraging the expression of warmth, concern, and caring toward the self [62]. It has been hypothesized that this process promotes adaptive emotional responding by activating physiological systems associated with self-soothing and caregiving [47, 63].

Only two studies known to us have examined self-compassion in the context of Gratz and Roemer’s [54] multidimensional emotion regulation model; in these studies, a significant inverse correlation was found between self-compassion and emotion regulation difficulties among adults with generalized anxiety disorder [64] and among adolescents and young adults with a history of childhood maltreatment [65]. In the latter study, self-compassion was found to explain variation in problematic emotion regulation over and above other risk factors such as severity of childhood maltreatment history, current psychological distress, and addiction history [65]. Other studies exploring the association between self-compassion and emotion regulation have reported negative links between self-compassion and specific maladaptive emotion regulation strategies, such as rumination [32, 50, 51], thought suppression [32], and avoidance [34, 51]. Self-compassion has also been found to positively predict coping in the face of difficult emotional experiences (e.g., [34, 35, 46]) and is linked to aspects of positive emotion regulation such as emotional clarity and emotional repair [32]. In line with this, self-compassion training has been hypothesized to moderate responses to social stress by promoting adaptive emotion regulation [47].

### Self-Compassion and Occupational Stress among Psychologists

Self-compassion has been investigated as an outcome variable in a small number of studies examining the impact of stress-reduction initiatives among health care professionals, which provide some insight into the potential for self-compassion to promote stress resilience (for a review, see [21]). For example, an early study by Shapiro, Astin (24) found that health care professionals undergoing MBSR training reported significant increases in self-compassion and decreases in stress relative to waitlist controls, and that reductions in stress were mediated by self-compassion. Similarly, a study by Shapiro, Brown (23), investigating the impact of MBSR training among master’s level trainee counselling psychologists found that post-test increases in self-compassion co-emerged with decreases in perceived stress, negative affect, rumination, and anxiety; however, self-compassion was not investigated as a mediating variable in this study. More recently, qualitative studies have highlighted the relevance of self-compassion in managing stress among counsellors [30] and trainee therapists [66]. Despite the methodological limitations noted across the studies they reviewed, Boellinghaus, Jones (21) concluded that interventions that foster the development of self-compassion among clinicians have the potential to promote wellbeing and reduce stress-related outcomes such as empathic distress and burnout.

The relevance of self-compassion for psychologists can be further understood in the context of the unique challenges faced by this occupational group as part of their professional role. Psychologists regularly bear witness to others’ emotional distress, with research suggesting that routinely relating to suffering in an empathic way can lead to increased negative affect and shared feelings of distress [67], compassion fatigue [9], and secondary traumatization [11, 68]. Psychotherapeutic work also involves learning to tolerate ambiguity in symptom presentation, and to manage multiple aspects of practitioner competence to establish and maintain effective therapeutic relationships [69]. In addition, psychologists report specific types of challenges presented by working with clients who suffer from personality disorders [70], those who are resistant to treatment or drop out of therapy prematurely [71], and those who are suicidal [72, 73]. Working with such clients may evoke strong feelings of hopelessness, inadequacy, self-doubt, grief, and fear among clinicians and cause them to question their own professional competence [70, 74, 75]. These experiences may be particularly problematic for psychologists have high performance expectations [76] or who routinely minimize their own needs and neglect self-care [19, 31, 77]. In light of these findings, we believe that self-compassion may represent an important resource that helps psychologists respond to the challenges of therapeutic work in an emotionally balanced way, thereby reducing their experience of stress.

### Aims of the Present Study

The aim of the present study was to clarify the link between self-compassion and stress among professional and trainee psychologists in Australia. As there is no dominant model that accounts for the relationship between self-compassion and the markers of psychopathology with which it is consistently negatively associated, we sought to advance the theoretical understanding of the mechanisms involved in the self-compassion-stress relationship by testing an explanatory model of self-compassion. Based on initial evidence that self-compassion shares strong, negative links with emotion regulation difficulties [65, 78], which, may in turn impact stress [47] and other symptoms of psychopathology [52], the present study examined the mediating role of emotion regulation difficulties in the relationship between self-compassion and stress symptoms.

We sought to explore the relationship between these variables after controlling for neuroticism. Neuroticism is a personality variable found to consistently influence individuals’ experience of, and response to, stress (e.g., [79, 80, 81]), and it has been proposed that psychology trainees who are high in neuroticism may experience enduring difficulties adjusting to the stressors of clinical training [82]. Given that the tendency to ruminate, feel isolated, and criticize oneself is characteristic of both low self-compassion and high neuroticism [33], it is important to account for the influence of neuroticism in the relationship between self-compassion and stress. While Neff, Rude (33) found that self-compassion predicted variance in positive psychological functioning after the influence of personality factors (including neuroticism) were controlled, previous research investigating the relationship between self-compassion and various psychological distress outcomes has been limited in that the influence of neuroticism has not been accounted for.

**Fig 1:**
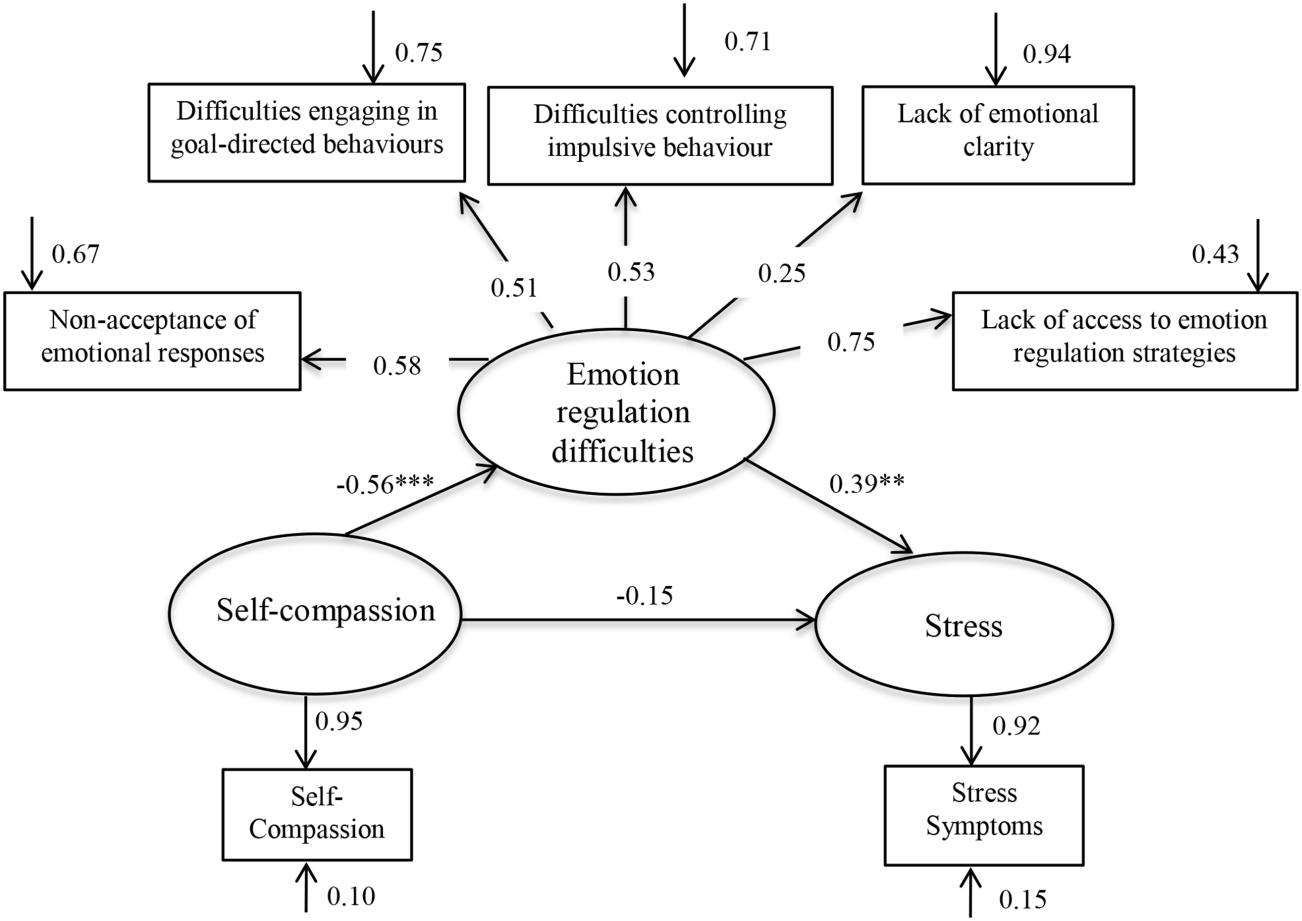
Parameter estimates for the partial mediator model. Statistical significance ** *p* < .01, *** *p* < .001.

**Fig 2:**
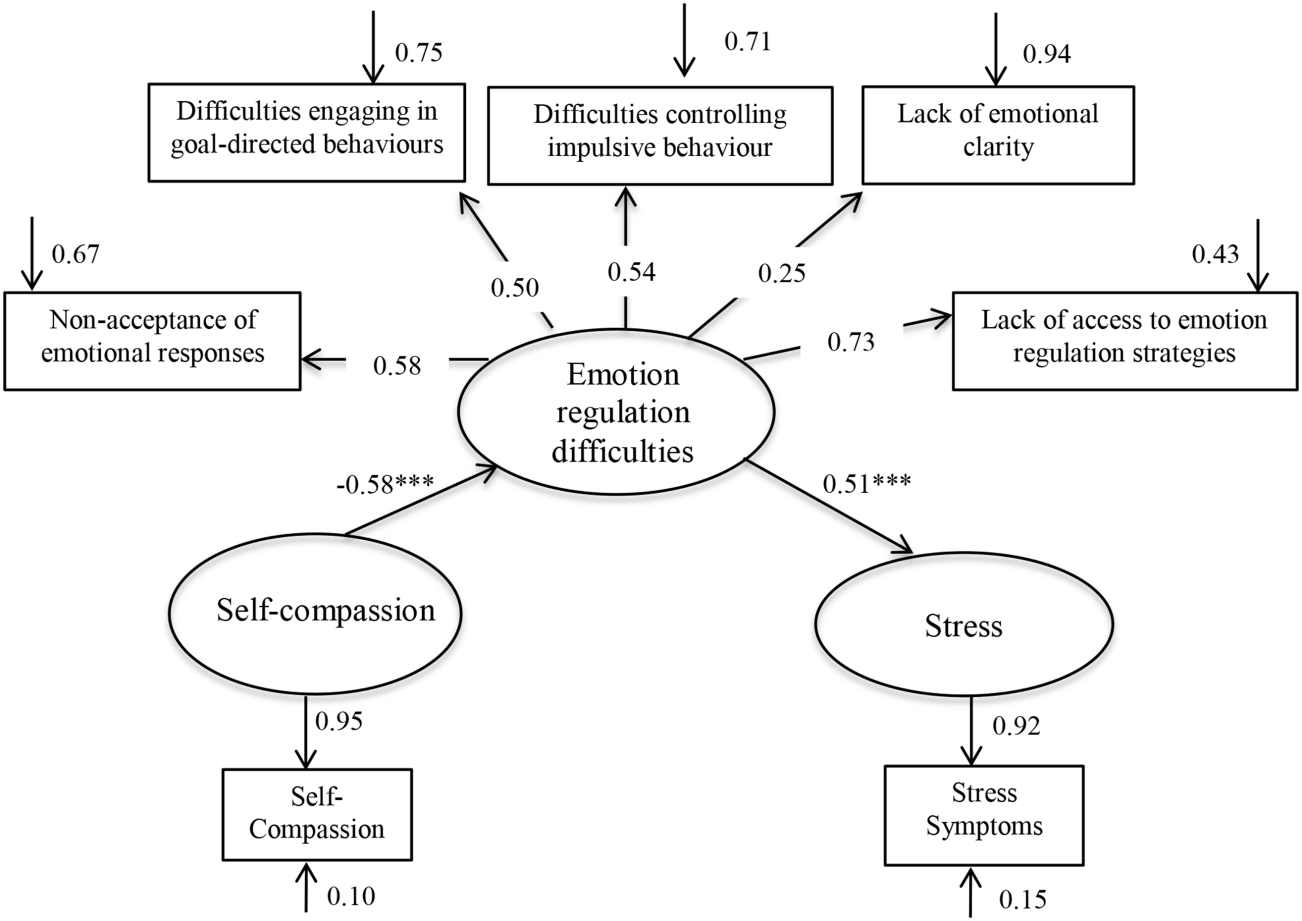
Parameter estimates for the full mediator model. Statistical significance *** *p* < .001.

We hypothesized that after controlling for neuroticism: (1) self-compassion would negatively predict stress symptoms (2) self-compassion would negatively predict emotion regulation difficulties; (3) emotion regulation difficulties would positively predict stress symptoms; and (4) when the variables were examined in the context of a structural equation model, the data would support a model in which emotion regulation difficulties mediate the relationship between self-compassion and stress. Given that this is a preliminary investigation of the relationship between these variables, we did not hypothesize whether emotion regulation difficulties would partially or fully mediate the self-compassion-stress link. Instead, an exploratory examination of a full mediation model was conducted by comparing the model fits for a partial mediation model (Fig 1) and a full mediation model (Fig 2).

## Method

### Ethics Statement

Ethical approval for this study was granted by the Curtin University Human Research Ethics Committee.

### Participants

Participants were 27 male and 171 female Australian psychologists who were currently engaged in clinical work. Of these 125 were “trainees”—students currently enrolled in a post-graduate clinical or counselling psychology degree, while 73 were “practising psychologists”—non-students who identified psychology as their primary profession. To be included in the study, participants had to self-identify as a provisional or full member of the Australian Health Practitioner Regulation Agency (AHPRA) the regulating body for health practitioners in Australia. While the gender distribution in the current study was skewed (86% female), it should be noted that estimates of the ratio of females to males within the workforce of Australia psychologists is around 80:20 [83].

### Latent Variables and Measures

#### Demographic questionnaire

Participants completed a demographic questionnaire that assessed age, gender, current occupation (trainee or practising psychologists), highest education degree attained, duration of clinical experience, and average number of hours spent in clinical practice per week. In addition, trainee psychologists were asked about the type and stream of their current degree. Practising psychologists were asked to specify their primary profession.

#### Self-compassion

The Self-Compassion Scale-Short Form (SCS-SF [84]) is a 12-item self-report measure used to measure trait self-compassion. This measure employs a five-point Likert-type response format, ranging from 1 (*almost never*) to 5 (*almost always*). The SCS-SF is a short version of the 26-item Self-Compassion Scale [32], and measures self-compassion across three dimensions self-kindness (e.g., “*I try to be understanding and patient towards those aspects of my personality I don’t like”*), common humanity (e.g. “*I try to see my failings as part of the human condition*.”), and mindfulness (e.g. “*[w]hen something painful happens I try to take a balanced view of the situation*”). The inverse of these dimensions is also assessed using self-judgment, isolation, and over-identification subscales, with items from these scales reverse-scored. When examining total scores, the SCS-SF correlates highly with the long scale, which has strong internal consistency, test-retest reliability, and convergent and discriminant validity [84]. The SCS-SF has demonstrated adequate internal consistency [Cronbach’s alpha ≥ .86; 84].

#### Emotion regulation difficulties

The Difficulties in Emotion Regulation Scale (DERS, [54]) is a 36-item measure designed to assess difficulties in emotion regulation using a five-point Likert-type response format (1 = *almost never* to 5 = *almost always*). Emotion regulation difficulties are measured along six dimensions: (1) non-acceptance of emotions (e.g., “*[w]hen I’m upset I become angry with myself for feeling that way*”); (2) difficulties engaging in goal-directed behaviour when upset (e.g. “*[w]hen I’m upset I have difficulty focusing on other things*”); (3) impulse control difficulties when upset (e.g., “[*w]hen I’m upset I have difficulty controlling my behaviours*”); (4) lack of emotional awareness (e.g., “*I pay attention to how I feel*” [reverse scored]); (5) limited access to emotion regulation strategies (e.g. “*[w]hen I’m upset*, *I believe that wallowing in it is all I can do*”; and (6) lack of emotional clarity (e.g., “*I have difficulty making sense out of my feelings*”). Although the DERS has exhibited good validity, test-retest reliability, overall internal consistency (α = .93) and adequate subscale reliability, [54, 85], recent evidence indicates that the *Awareness* dimension may not represent the same higher-order emotion regulation construct as the other five dimensions [86]. As a result, the Awareness subscale was not used as an indicator of the latent Emotion Regulation Difficulties construct in the SEM analysis.

#### Stress symptoms

Participants completed the full 21-item version of the Depression Anxiety Stress Scales [87], however only scores on the stress subscale were used in the data analysis, as a measure of psychobiological symptoms of stress. The DASS-21 was developed to measure symptoms of depression, anxiety, and stress in clinical and non-clinical populations. The stress subscale contains 7 items that assess how often in the past week respondents experienced stress symptoms—i.e., symptoms of chronic, non-specific arousal, such as nervous tension, difficulty relaxing, and a tendency to be irritable, impatient, and easily agitated. It employs a four-point Likert-type response format, with possible responses ranging from 0 (*did not apply to me at all*) to 3 (*applied to me very much*, *or most the time*). The reliability of the stress subscale has previously been reported as .91 [88].

#### Neuroticism

Neuroticism was measured using the Neuroticism subscale of the Big Five Inventory (BFI [89]). The BFI is a 44-item questionnaire that assesses the Big Five Personality domains. Each item is a short descriptive statement, and respondents are asked to rate how much the characteristics described applies to them, using a 5-point Likert-type response scale (1 = *disagree strongly* to *5* = agree strongly). The BFI has been found to have good reliability, a clear factor structure, convergent validity with longer Big Five measures, and adequate self-peer agreement [90].

### Procedure

Participants were recruited via direct email, through professional psychology bodies and universities with postgraduate psychology programs around Australia, as well as through social media. Participants provided written consent online and completed all measures online using Qualtrics, with administration of measures randomised to reduce order effects.

## Results

### Preliminary Analyses and Descriptive Statistics

The demographic characteristics of the sample are presented in S1 Table. The means, standard deviations, ranges, and internal consistency reliability estimates for the 7 observed variables are reported in S2 Table. As the 7 observed variables were not multivariate normal, Spearman’s correlation coefficients were used as an index of correlation amongst these variables [91]. These correlations are also reported in S2 Table.

Prior to testing the structural equation models, confirmatory factor analysis (CFA) was used to determine the extent to which the observed variables measured the latent constructs. The results of the CFA conducted on the Stress scale items of the DASS-21 supported the stress scale as a single-factor construct. In addition, a 1-factor solution was confirmed for self-compassion. For each single-indicator latent variable, the error variance was computed by subtracting the reliability of the scale used to measure the indicator from 1.

A CFA was conducted for the latent emotion regulation difficulties variable to investigate an emotion regulation difficulties construct with 5 indicators. In line with previous findings [86], a 5 factor emotion regulation difficulties construct based on the non-acceptance, strategies, clarity, impulse control and goal direction subscales of the DERS was supported by the data.

Structural equation modeling with maximum likelihood estimation was used to determine the extent to which emotion regulation difficulties mediated the relationship between self-compassion and stress. The analyses were carried out using LISREL version 9.10 [92]; as the variables were not multivariate normal, all tests were performed using Browne’s [93] ADF chi-square, which does not assume multivariate normality [94]. Several indicators were used to evaluate model fit; good fit was indicated by the following cut-off values: less than 3 for χ^2^/*df [95]*, above .90 for the comparative fit index (CFI) and the non-normed fit index (NNFI; [96]), below .10 for the standardized root-mean-square residual, and below .05, or a confidence interval that encompasses this value, for the root-mean-square error of approximation [97]. All of the SEM tests met Kline’s [95] suggested minimum cases-to-parameter ratio of 5:1.

### Control Variables

Potential control variables included age, gender, years of experience, and occupational group (i.e. practising or trainee psychologist), and neuroticism. Significant bivariate correlations were found between age and years of experience and each of the indicator variables, however a linear regression with age and years of experience as predictors revealed that the relationship between years of experience and the indicator variables were non-significant once age was controlled for. As a result, age was retained as a control variable, whereas years of experience was not. Significant bivariate correlations were also found between neuroticism and each of the indicator variables: as a result, neuroticism was also controlled in the current study.

To determine whether the partial correlations among the nine indicators varied as a function of occupational group (i.e. professional versus trainee psychologists) or gender, we conducted a multi-group analysis of the equality of the correlation matrices, based on the partial correlations between the variables after controlling for age. The relationship among the indicators did not vary as a function of occupational group, χ^2^ (45, *N* = 198) = 29.33, *p* = 0.97 or gender χ^2^ (55, *N* = 198) = 17.27, *p* = 0.99. As a result, it was considered appropriate to collapse the data across occupational group and gender.

### LISREL Analyses

#### Test of the measurement model

Confirmatory factor analysis indicated that the initial measurement model showed an adequate fit to the data, suggesting that it was appropriate to test the structural model. The fit statistics for the measurement model are given in S3 Table.

The correlations amongst the full set of latent variables are shown in S4 Table. In support of Hypotheses 1 and 2, significant negative correlations were observed between self-compassion and emotion regulation difficulties, -.56, *p* < .001 and self-compassion and stress, -.36, *p* < .001). In support of Hypothesis 3, significant positive correlations were observed between emotion regulation difficulties and stress, .48, *p* < .001. These findings satisfied the first three requirements of Baron and Kenny’s [98] four step analytical procedure for testing mediation models.

#### Test of the partial mediation model

The partial mediation model with its parameter estimates is shown in Fig 1. In this model, the pathway from self-compassion to stress was non-significant, indicating that there was no direct pathway from self-compassion to stress once emotion regulation difficulties, neuroticism and age were controlled. All other pathways were significant. This finding provides support for Hypothesis 4. The fit indices for the partial mediation model suggested an adequate fit to the data (see S3 Table).

#### Test of the full mediation model

The full mediation model with its parameter estimates is shown in Fig 2. The fit indices for the full mediation model suggested an adequate fit to the data (see S3 Table). A chi-square difference test was performed to determine whether the partial mediation model (with one extra pathway) fit the data significantly better than the full mediation model. The result of this test was non-significant χ^2^_difference_ (1, *N* = 198) = 1.6, *p* = .21, suggesting that the data supported a model in which the relationship between self-compassion and stress was fully mediated by emotion regulation difficulties. The final model accounted for 34.0% of the variance in emotion regulation difficulties, and 26.2% of the variance in stress symptoms.

#### Test of the indirect effects

A Sobel test was used to determine whether the indirect pathway from self-compassion to stress via emotion regulation difficulties was significant. The results of this test suggested that the association between self-compassion and stress is significantly mediated by emotion regulation difficulties (z’ = 3.83, p < 0.001).

## Discussion

As health care paradigms expand to include the wellbeing of the clinician as a central consideration, self-compassion has been identified as an important target variable for researchers interested in preventing occupational stress-related conditions among psychologists [21, 66]. However, few studies have quantified or elucidated the relationship between self-compassion and stress outcomes among this occupational group. Across other populations, research that supports self-compassion as a predictor of a range of mental health outcomes has accumulated with limited insight into the underlying processes involved in these relationships. The aim of the current study was to advance the understanding of the utility of the self-compassion construct in promoting stress resilience among trainee and professional psychologists in Australia, as well as to provide some clarity regarding the relationship between self-compassion and stress more generally. After controlling for age and neuroticism, self-compassion was found to be a significant predictor of stress symptoms, with emotion regulation difficulties mediating this relationship. In SEM terms, self-compassion did not have a direct impact on stress symptoms; rather, it impacted stress symptoms via a reduction in emotion regulation difficulties.

The final model accounted for 26.2% of the variance in stress, adding to the evidence supporting self-compassion as a reliable predictor of stress [for a review, see 40]. A notable finding in the current study was that the relationship between self-compassion, and emotion regulation difficulties and stress was significant once age and neuroticism were controlled for. Research has consistently found that neuroticism is positively related to emotion regulation difficulties [99], stress [100, 101], and more specific occupational stress outcomes, such as burnout [102]. Inclusion of neuroticism as a covariate therefore allowed us to address the alternative hypothesis the relationship between self-compassion, emotion regulation difficulties, and stress is better explained by the common influence of neuroticism. This represents an important contribution to the literature, given that the majority of work examining the relationship between self-compassion and psychological health has failed to take neuroticism into account. In addition, these findings add to literature suggesting that self-compassion may represent an important intervention target in programs designed to promote stress resilience among psychologists and other health professionals [2, 25, 66].

The current study also extends the literature documenting the link between self-compassion and emotion regulation difficulties [50, 65, 78], and indicates that the relationship between self-compassion and stress can be understood in terms of a mechanism by which self-compassion indirectly impacts stress via a reduction in emotion regulation difficulties. These findings suggest that individuals who are more self-compassionate experience more emotional clarity and are more accepting of difficult emotions. In addition, they are less likely to experience difficulties controlling impulsive behavior in the face of stressful experiences, and are more able to access effective emotion regulation strategies, thereby promoting adaptive responding to stress. Previous research has found that rumination and worry—two known maladaptive emotion regulation strategies—mediate the relationship between self-compassion and symptoms of depression and anxiety [50, 51]. Additionally, in a randomized controlled trial of and 8-week Mindful Self-Compassion program, Neff and Germer [103] found that significant reductions in avoidance accompanied reductions in stress among the intervention group. In the context of these findings, the results of the current study may be extrapolated to suggest that difficulties with emotion regulation may represent a key explanatory mechanism underlying the link between self-compassion and psychological health more broadly.

According to emotion regulation theory, it is one’s ability to regulate the *severity* and *duration* of emotional responses, rather than alter the *type* of emotion experienced that promotes adaptive responding in the face of stress [104]. Self-compassion is thought to promote a balanced perspective of negative emotions, allowing individuals to confront difficult feelings and thoughts, rather than avoiding them or becoming entrenched in them [62]. Individuals who are higher in self-compassion are more likely to think about negative events in ways that are adaptive: they are more objective, less likely to catastrophize and judge themselves harshly, and more able to see difficult experiences as a normal part of life [35, 46]. Moreover, responding to oneself with self-compassion involves actively soothing oneself rather than engaging in self-criticism or self-blame in the face of stressful events [105, 106]. Hypothetically, responding to oneself in this way reduces the level of threat posed by a specific stressor, and frees up self-regulatory resources so that the individual is able to cope more effectively with the situation at hand [107]. While some researchers have conceptualised self-compassion as an emotion regulation strategy in and of itself [44, 108], it is important to note that self-compassion is a process of self-to-self relation, rather than a process that focuses solely on one’s emotions [103, 109]. Along these lines, we argue that self-compassion represents a self-regulatory process that gives rise to adaptive emotion regulation, among other positive outcomes.

Self-compassion is thought to be particularly useful when it comes to negative self-relevant events [46], as it promotes a sense of self-acceptance and positive self-worth that is independent of performance outcomes. In this sense, self-compassion has been identified as a more stable predictor of wellbeing than self-esteem [110]. These findings have important implications for psychologists, whose experience of occupational stress may center on interactions with “difficult” clients and is often characterised by feelings of self-doubt, inadequacy and performance anxiety [31, 69, 70]. We believe that psychologists who are more self-compassionate are less likely to base their personal or professional self-worth on positive therapeutic outcomes or favourable reactions from clients. As a result, they are more inclined to view the challenges of therapeutic work as inherent part of their professional role, rather than as an indication of their own failure or inadequacy. It may therefore be surmised that self-compassion helps to reduce therapists’ feelings of apprehension, self-consciousness and self-rumination both within and outside of the therapeutic hour [110]. This outcome may be particularly important for trainees [111, 112], and suggests that self-compassion training may be an important addition to clinical training programs.

In the context of previous findings that self-compassion positively predict aspects of emotional intelligence such as emotional clarity and mood regulation [32, 113], the current findings support the proposition that therapists who are more self-compassionate are likely to be more mindful of stressful work-related experiences and difficult emotional states, kinder to themselves when they receive negative or ambiguous feedback, and more likely to utilize adaptive emotion regulation strategies and self-care strategies during or following stressful encounters [30, 66, 114]. Additionally, we propose that the sense of common humanity engendered by the self-compassionate mindset may work to defuse reactivity to challenging clients by promoting a sense of interconnectedness, reduce therapists’ feelings of isolation in their professional role, and support the understanding that developing as a psychologist involves making mistakes and experiencing setbacks [21]. Furthermore, it has been argued that self-compassion promotes decoupling of the relationship between taking responsibility for unpleasant self-relevant events and experiencing negative affect [46]. As a result, psychologists who are more self-compassionate may be more able to view difficulties and setbacks in a balanced way, which in turn supports the capacity to work through challenges effectively and use them as an opportunity for growth [34]. Further research is recommended to explore these propositions, and to investigate how self-compassion training may impact psychologists’ responses to the stressors of clinical training and professional practice.

### Limitations and Future Directions

The current study offers a number of valuable insights into the relationship between self-compassion, emotion regulation difficulties, and stress; however, certain limitations should be noted. First, while stress symptoms generally precede more severe distress, burnout, and professional impairment [76, 115, 116], they should not be seen as equivalent to or necessarily predictive of these outcomes. Future research in this area may wish to consider the relevance of self-compassion to specific occupational health outcomes such as compassion fatigue and burnout. In addition, as self-compassion has consistently been found to negatively predict both depression and anxiety [40], it is recommended that the emotion regulation model of self-compassion proposed here be tested in relation to these outcomes. Further research in this area may also explore whether certain types of emotion regulation difficulties appear to be more relevant (i.e., account for more variance in outcomes) across different psychological disorders.

Due to the cross-sectional research design, the results of the current research cannot reliably address questions of causality between self-compassion and stress. While increased self-compassion may buffer against stress [117], it is also viable that lower levels of stress support a more self-compassionate perspective [42]. In order to address the question of causation, future research would benefit from the use of an experimental design to investigate the impact of changing self-compassion levels over time. As previous research indicates that self-compassion can be cultivated through training (see [103, 118, 119]), investigation of the effectiveness of self-compassion interventions for promoting resilience to stress among psychologists is an exciting direction for future research. In light of the results of the current study, previous findings documenting the link between self-compassion and emotion regulation difficulties [50, 65, 78, 103] and research highlighting emotion regulation as a key change process in acceptance- and mindfulness-based therapies (e.g.[57]), we recommend that the evaluation of such interventions take emotion regulation into account when considering mechanisms of action. Finally in the context of recent research has found that self-compassion training enhances compassion for others [103] and increases the experience of positive affective states when witnessing others’ distress [120], it is worthwhile considering the impact of self-compassion training for psychologists on positive outcomes, such as compassion, therapeutic effectiveness and psychological wellbeing.

## Conclusion

There is growing evidence demonstrating self-compassion as a reliable predictor of psychological symptoms, including stress, anxiety and depression [42]. The current study adds to these findings by revealing significant links between self-compassion, stress, and emotion regulation difficulties in a sample of trainee and practising psychologists. Initial support was found for our emotion regulation model of self-compassion, however future research with other samples and a broader range of outcome criteria is needed to further test the generalizability of the model. In addition, the results from the current study support the proposition that the cultivation of self-compassion is an important target for interventions that aim to prevent or reduce occupational stress among this professional group [21].

## Supporting Information

S1 Table Participants’ Age, Gender, and Education. (DOCX)

S2 Table Means, Standard Deviations, Internal Consistency, and Spearman’s Correlations for Observed Variables. Statistical significance ** *p* < .01, *** *p* < .001; SCS-SF: Self-Compassion Scale-Short Form; DERS: Difficulties with Emotion Regulation Scale; DASS-21: 21-Item Depression, Anxiety, Stress Scales.
(DOCX)

S3 Table Fit Statistics for Structural Equation Models. *χ* ^2^/df: A value greater less than 3 indicates a good fit (Kline, 2005); CFI, Comparative Fit Index: A value greater than or equal to .90 indicates a good fit (Hu & Bentler, 1999). NNFI, Non Normed Fit Index: A value greater than or equal to .90 indicates a good fit (Hu & Bentler, 1999); SRMR, Standardized Root Mean Square Residual: A value less than or equal to .08 indicates a good fit (Hu & Bentler, 1999); RMSEA, Root Mean Square Error of Approximation: A value less than or equal to .05, or a CI that encompass this value, indicates a good fit (Jaccard & Wan, 1996).
(DOCX)

S4 Table Intercorrelations Among Latent Variables. Statistical significance: *** *p* < .001.
(DOCX)
