# Supplementary material for: Self-Compassion, Emotion Regulation and Stress among Australian Psychologists: Testing an Emotion Regulation Model of Self-Compassion Using Structural Equation Modeling
Source: PLoS One. 2015 Jul 24;10(7):e0133481. doi: 10.1371/journal.pone.0133481 (PMC4514830; doi:10.1371/journal.pone.0133481)
Supplement: S1 Table — (DOCX) [file pone.0133481.s001.docx]

| Table 1. Participants’ Age, Gender, and Education. | | |
| --- | --- | --- |
|  | **Mean** | ***SD*** |
| Age | 36.25 | 11.79 |
|  | **Frequency** | **Percentage** |
| Gender  Male  Female  Current Occupation  Trainee Psychologist  Practising Psychologist  Education Level  Bachelor Degree  Graduate Diploma  Post-Graduate Diploma  Master’s Degree  PhD/Doctorate | 27  171  105  93  85  8  17  60  27 | 13.60  86.40  53.00  47.00  42.9  4.0  8.6  30.3  13.6 |
