# Supplementary material for: Self-Compassion, Emotion Regulation and Stress among Australian Psychologists: Testing an Emotion Regulation Model of Self-Compassion Using Structural Equation Modeling
Source: PLoS One. 2015 Jul 24;10(7):e0133481. doi: 10.1371/journal.pone.0133481 (PMC4514830; doi:10.1371/journal.pone.0133481)
Supplement: S2 Table — Statistical significance ** p < .01, *** p < .001; SCS-SF: Self-Compassion Scale-Short Form; DERS: Difficulties with Emotion Regulation Scale; DASS-21: 21-Item Depression, Anxiety, Stress Scales. (DOCX) [file pone.0133481.s002.docx]

| **Table 2.**  Means, Standard Deviations, Internal Consistency, and Spearman’s Correlations for Observed Variables. | | | | | | | | | | |
| --- | --- | --- | --- | --- | --- | --- | --- | --- | --- | --- |
| **Variable** | **1** | **2a** | **2b** | **2c** | **2d** | **2e** | **3** | ***M*** | ***SD*** | ***α*** |
| 1. SCS-SF | - |  |  |  |  |  |  | 3.27 | 0.74 | 0.90 |
| 2a. DERS Non-Acceptance | -.58*** | - |  |  |  |  |  | 11.20 | 4.91 | .91 |
| 2b.DERS Goal Direction | -.47*** | .32*** | - |  |  |  |  | 13.21 | 4.58 | .90 |
| 2c. DERS Impulse Control | -.55*** | .50*** | .46*** | - |  |  |  | 9.15 | 3.00 | .78 |
| 2d. DERS Strategies | -.69*** | .59*** | .55*** | .62*** | - |  |  | 13.28 | 5.01 | .89 |
| 2e. DERS Clarity | -.29*** | .29*** | .22** | .32*** | .28*** | - |  | 8.80 | 2.51 | .75 |
| 3. DASS-21 Stress | -.55*** | .51*** | .38** | .50*** | .60*** | .21** | - | 11.96 | 8.00 | .85 |
