# Supplementary material for: Self-Compassion, Emotion Regulation and Stress among Australian Psychologists: Testing an Emotion Regulation Model of Self-Compassion Using Structural Equation Modeling
Source: PLoS One. 2015 Jul 24;10(7):e0133481. doi: 10.1371/journal.pone.0133481 (PMC4514830; doi:10.1371/journal.pone.0133481)
Supplement: S3 Table — χ 2/df: A value greater less than 3 indicates a good fit (Kline, 2005); CFI, Comparative Fit Index: A value greater than or equal to .90 indicates a good fit (Hu & Bentler, 1999). NNFI, Non Normed Fit Index: A value greater than or equal to .90 indicates a good fit (Hu & Bentler, 1999); SRMR, Standardized Root Mean Square Residual: A value less than or equal to .08 indicates a good fit (Hu & Bentler, 1999); RMSEA, Root Mean Square Error of Approximation: A value less than or equal to .05, or a CI that encompass this value, indicates a good fit (Jaccard & Wan, 1996). (DOCX) [file pone.0133481.s003.docx]

| **Table 3.** Fit Statistics for Structural Equation Models | | | | | | |
| --- | --- | --- | --- | --- | --- | --- |
| **Model** | ***χ*^2^/df** | **CFI** | **NNFI** | **SRMR** | **RMSEA** | **Model AIC** |
| Measurement Model | 30.40/13 = 2.34 | .94 | .91 | .05 | .08 (90% CI: .05, .12) | 1203.98 |
| Saturated Structural Model | 30.40/13 = 2.34 | .94 | .91 | .05 | .08 (90% CI: .05, .12) | 1203.98 |
| Mediation Model | 32.00/14 = 2.28 | .94 | .90 | .05 | .08 (90% CI: .05, .12) | 1204.22 |
