# Supplementary material for: Self-Compassion, Emotion Regulation and Stress among Australian Psychologists: Testing an Emotion Regulation Model of Self-Compassion Using Structural Equation Modeling
Source: PLoS One. 2015 Jul 24;10(7):e0133481. doi: 10.1371/journal.pone.0133481 (PMC4514830; doi:10.1371/journal.pone.0133481)
Supplement: S4 Table — Statistical significance: *** p < .001. (DOCX) [file pone.0133481.s004.docx]

| **Table 4.** Intercorrelations Among Latent Variables | | | | |
| --- | --- | --- | --- | --- |
| Construct | 1. | 2. | 3. | 4. |
| 1. Self-Compassion | - |  |  |  |
| 2. Emotion Regulation Difficulties | -.56*** | - | - |  |
| 3. Stress Symptoms | -.37*** | .48*** | - | - |
